# International Expert Panel Consensus Guidelines for Structure and Delivery of Qigong Exercise for Cancer Care Programming

**DOI:** 10.3390/medicines4030054

**Published:** 2017-07-14

**Authors:** Penelope Klein, George Picard, Roger Schneider, Byeongsang Oh

**Affiliations:** 1 D’Youville College, Buffalo, NY 14201, USA; 2 Niagara College, St Catharines, ON L2R 7R3, Canada; george@georgepicard.com (G.P.); rschnei487@aol.com (R.S.); 3 Village of Healing, St Catharines, ON L2R 7R1, Canada; 4 Northern Sidney Cancer Center, Sydney, NSW 2065, Australia; byeong.oh@sydney.edu.au

**Keywords:** Qigong, consensus guideline, cancer, supportive care, integrative medicine

## Abstract

Integrative oncology, including Qigong, is a relatively new concept in modern healthcare. Evidence of benefits of Qigong in cancer survivors is emerging. As such, several cancer centers, world-wide, have introduced Qigong as part of integrative medicine within supportive cancer care programming. Qigong exercise programming content and quality varies among institutions due to lack of standard guidelines and, at present, relies solely on the instructor’s skills, knowledge, personal preferences and clinical experience. Development of consensus guidelines recommending the basic structure and delivery of Qigong programming in cancer care can potentiate quality assurance and reduce risk of harm. This applied qualitative research utilized a modified Delphi approach to formulate consensus guidelines. Guidelines were developed through discussions among an international expert panel (*N* = 13) with representation from Australia, Canada, Ireland, and the United States. Panel communication was predominantly conducted by email and occurred from November 2016 through February 2017. Expert panel work resulted in the generation of a work product: Qigong in Cancer Care Guidelines: A Working Paper including: (a) Consensus Guidelines for structure and delivery of Qigong exercise for Cancer care programming; (b) Consensus guidelines for instructor competence for teaching Qigong exercise for cancer care classes; (c) Screening tool for safe participation in Qigong exercise; (d) Class participant instructions for maintaining safety during Qigong exercise; and (e) Advice from the field. Generation of these resources is the first step in establishing recommendations for ‘best practice’ in the area of Qigong for cancer care programming.

## 1. Introduction

Cancer is pandemic. There are an estimated 15 million cancer survivors in the United States [1]. Additionally, approximately 15 million new cases are diagnosed, annually, throughout the world [2]. Qigong exercise, a mind/body exercise regimen encompassing gentle exercise, meditation, mind adjustment, and breath regulation has been an integral component of traditional Chinese medicine (TCM) for millennia [3]. Recent scientific research has validated that, among its many therapeutic applications, Qigong exercise can improve cancer-related quality of life (QOL) [4,5]. In a recently released report of a 24-week, randomized clinical trial with active controls, Liu et al. assessed effects of Goulin-Qigong (GLQC) on 158 subjects with cancer [6]. Comparative analysis found that both GLQG and physical stretching are beneficial during recovery following breast cancer. However, GLQC was more effective in terms of QOL improvements. With regard to comparison of GLQC vs. a program of physical exercise, both regimens resulted in comparable improvements in anxiety or depression. However, the GLQC practice had a greater effect on immunological function. This research confirms therapeutic effect of Qigong practice and demonstrates superior effect as compared to traditional exercise modes with respect to QOL and immunilogical outcomes.

In China, Qigong exercise is widely practiced in management of cancer in conjunction with standard care. Applied theoretical research suggests that practice of Qigong exercise may also have a supportive role in cancer prevention and improved survival [7,8,9]. However, additional large-scale population studies are needed to fully assess these associations. Further, Qigong practice has been found to be without serious adverse consequence [10] and to be cost-effective in targeted clinical applications [11,12]. However, wide-spread knowledge of this ancient, health-promoting modality is not common in the West.

Recently, the Office of Cancer Complementary and Alternative Medicine of the National Cancer Institute (NCI) released proceedings from its Strategy Meeting for the Development of an International Consortium for Chinese Medicine [13]. Among goals of that work was to better inform medical communities of the potential benefits of Qigong for individuals with chronic disease including cancer. In a similar effort to inform, the Society for Integrative Oncology (SIO), an international organization dedicated to encouraging scientific evaluation and integration of complementary therapies based on evidence, recommended Qigong in their evidence-based clinical practice guidelines for breast cancer. Albeit, they listed strength of evidence cited as weak [14]. Among others, Dana-Farber Cancer Institute, MD Anderson Cancer Center, Memorial Sloan-Kettering Cancer Center, and cancer centers in Europe and Australia have introduced Qigong within their supportive cancer care programming. Authority endorsement, growing research evidence, and awareness of positive experiences within pioneering institutions is creating a growing demand for Qigong programming in both primary care and community-based, wellness cancer centers. 

Acupuncture consensus statements and guidelines for safe practice in cancer survivors have been developed by the National Institutes of Health (NIH) in USA [15] and in the UK [16]. No comparable guidelines for delivery and structure of Qigong exercise in cancer care exist. A few national and international, not-for-profit organizations as well as diverse, independent business entities offer various types of Qigong instructor training and certification. Styles, forms, underlying philosophy, and requirements of training vary, for there may be as many styles of Qigong as there are Qigong masters. Thus structure of and content of existing Qigong programming being offered in cancer care centers relies on individual instructor knowledge, skills, clinical experience, and personal preferences to determine programming content and structure. In light of this fact and embracing the caveat that no one Qigong system is necessarily superior to others, a panel of international Qigong experts with knowledge and experience in serving individuals with cancer was convened. The vision of this undertaking was to advance availability and quality of Qigong exercise programming as a therapeutic modality within Integrative Oncology. The specific purpose of the panel work was to initiate development of guidelines to assist in planning, delivery, and staffing of quality Qigong in cancer care programming. The work is limited to care practiced within settings following a model of modern Western medicine and does not represent care as practiced within a TCM model. It should serve as a resource for Qigong instructors who are just entering practice in this area, decision-makers with influence on administration of Qigong in cancer care programming, and educators who train instructors. Herein, the methods and resultant products of this work are presented.

## 2. Materials and Methods 

This applied qualitative research utilized a modified Delphi approach to formulate a consensus draft of best-practice guidelines for structure and delivery of complementary Qigong exercise in cancer care. An international expert panel of convenience was assembled to conduct the work. Panel communication was predominantly conducted by email and occurred from November 2016 through February 2017. 

### 2.1. Research Flow

Initially, draft guidelines were developed by a subcommittee of convening panel members. A full panel of experts was then assembled, and three rounds of panel discussion were initiated. In the first round, panel members independently reacted by comment to the draft guidelines. In the second round, panel members reacted to a collation of the independent panel comments generated from the first round. Then, an internal subcommittee, including the three originating members in collaboration with an additional panel member, serving as medical consultant, drafted revised guidelines reflecting and incorporating panel member comments from Rounds 1 and 2. In the third round, the proposed revised guidelines were circulated to poll level of panel member consensus. 

### 2.2. Panel Recruitment

In planning study design, several factors were considered. A target panel size of 12 to 15 experts was set. Panel members were to include researchers as well as practitioners. Practice settings were limited to those consistent with a delivery model of modern Western medicine. A wide geographic representation was considered to be desirable. A list of potential panel members was generated from instructors listed with the National Qigong Association and the Qigong Institute websites who indicated interest in cancer as a clinical teaching area, identification of Qigong instructors affiliated with cancer centers listing Qigong or Tai chi classes as part of support programming, authors of published research, and nomination by panel members. By this process, 20 experts were identified and contacted regarding interest in joining the panel. Thirteen experts consented to participate.

### 2.3. Tools

Three data gathering tools were employed in this study, a survey of panel demographics and existing program characteristics, a survey of agreement and comment on the draft guidelines, and a polling of consensus for the resultant revised guidelines.

The first survey, *Panel Member Demographics Survey* (and description of exiting programming) was administered in Round 1. This survey gathered information regarding panel composition by gender, age, geographic locale, credentials, research activity, Qigong/Tai chi training, years of practice, years of teaching, years teaching in Qigong in Cancer care programming, and descriptions of active Qigong in Cancer Care programming.

The second survey, *Review and Comment Survey* (of draft guidelines), was also administered in Round 1. Draft guidelines were prefaced by a philosophy statement. They were organized into three major sections: Content (7 items), Instructor Competence (18 items), and Delivery Logistics (6 items). Panel members reacted by (a) level of agreement per each individual draft guideline, and (b) comments solicited by item and major section. Level of agreement for each of the 31 recommendations was assessed by a 4-point, Likert-like scale using the categories: *strongly agree, agree, disagree,* and *strongly disagree*. Comment solicitation was open-ended. 

Final consensus polling occurred in Round 3. In this final round, a summary working paper including revised guidelines and three additional panel products was circulated. Polling of agreement was conducted by vote: *approve* or *disapprove* of the work product in total with opportunity for additional comment.

### 2.4. Protection of Human Subjects

This research was conducted with full IRB approval through D’Youville College, Buffalo, NY, USA and has been performed in accordance with the ethical standards as laid down in the 1964. Declaration of Helsinki and its later amendments or comparable ethical standards.

### 2.5. Statistical Analysis

Analyses were descriptive. Panel demographics and characteristics of existing programs were recorded and described for report presentation. Panel comments were categorized through thematic analyses. For determining consensus, a criterion of ≥80% approval of final products was set. Consensus products generated by this work were described as work products. Work products were then integrated into a summary *Working Paper*.

## 3. Results

### 3.1. Panel Demographics

The 13-member expert panel is described as follows. The panel included seven males and six females. Average age was 59.77 years (SD 7.143). There was international representation. Countries represented included Australia, Canada, Ireland, and the United States. Within the United States, members represented the Northeast, Southern Atlantic, Central, Midwestern and Western regions. Seven of the 13 members hold graduate academic degrees. Seven members have published research. Six members have published instructional books or DVD’s. Three hold clinical degrees including a doctor of Chinese Medicine, a medical doctor with additional certification in medical acupuncture, and a physiotherapist. Nine members were currently teaching in dedicated cancer-care. Five members professed intimate knowledge of the cancer experience. Two members were in active cancer treatment during the time the research was being conducted. One panel member withdrew during Round 2 discussions citing other commitments as reason for withdrawal.

Mean years-of-practice was reported as 20.5 years (SD 10.14; range 4.5−40.0 years). Mean years teaching was reported as 15.38 years (SD 9.5). Panel members have a collective total of 100 years experience teaching Qigong classes dedicated for cancer care. Member training and practice involves a wide range of styles and forms including Yang, Chen, Sun and other derivative Tai chi styles and various forms of Qigong and modern derivations of traditional Qigong. All members have training in more than one style and/or form. Two or more members have training in each of the following: 24 Posture Qigong (originated by Master Wang Ziping), eight Brocades (as taught by Dr. Yang Zwing Ming) ‘Moving for better balance’ (originated by Dr. Fuzong Li), Tai Chi for Arthritis (originated by Dr. Paul Lamb), Easy Qigong/Tai Chi™ (originated by Dr. Roger Janhke), and some variation of instruction in sitting Qigong/Tai chi. There was no formal training reported specific to teaching Qigong to individuals with cancer by any panel member.

### 3.2. Characteristics of Existing Qigong Exercise for Cancer Care Programming

Class organization for existing Qigong exercise classes (N = 9) submitted by panel members follows a similar pattern allowing for some fluidity of practice. Generally, there is a period of social interaction and then warm-up exercises that may include meditation and/or mindful exercise prior to main exercise portion of the class. The major class time is dedicated to some form of Qigong/Tai chi exercise with periods of low activity or rest to accommodate varying levels of exercise tolerance, as needed, and integration of self-massage and meditation practice. Classes generally conclude with a short, interactive period that might include any combination of social support, discussion of stress reduction strategies and/or opportunity for questions and answers and discussion, or a quiet period of meditation. Qigong education might include application of energy cultivation and/or information about the latest research on Qigong in cancer care. (See Table 1 for a summary of class time utilization for existing programming as surveyed.)

### 3.3. Comment Integration and Consensus Polling

Response rates for contact rounds were: Round 1: 13/13, 100%; Round 2: 12/13, 92.3%; and Round 3: 12/12, 100%. Twelve panelists completed the study. As noted in the Methods section of this report, one panel member withdrew during Round 2 citing other time commitments as reason for withdrawing. Consensus poll unanimously (12/12) approved the *Working Paper* and its included work products. 

In Round 1, there was strong panel approval of the initial draft guidelines with suggestions for minor revisions plus suggestions for additional content as well as informational and cautionary advice to prospective instructors. Subcommittee, comment collation, review and consideration of comments from Rounds 1 and 2 (For a full thematic collation of panel comments see Supplementary Material 1: Data) resulted in re-structuring of the initial 31-item, draft Guideline into two Guidelines: *Consensus recommendations for structure and delivery of Qigong exercise for Cancer care programming* (See Table 2) and *Consensus recommendations for instructor competence for teaching Qigong exercise for cancer care classes* (See Table 3). Also in response to panel comments, three additional products were generated by the 4-member panel subcommittee responsible for revising the guidelines into a cohesive final product. Dominant comment themes were program philosophy, class content, and knowledge of the instructor. A subtheme that crossed major themes was protection of a vulnerable population. Several comments were offered as advice to Qigong instructors who might be new to working with individuals with cancer. Additional resources generated through this work include (a) a screening tool for safe participation in Qigong exercise; (b) class participant instructions for maintaining safety during Qigong exercise; and (c) a listing of *Advice from the field*. All five products are presented within a summary *Qigong in Cancer Care Working Paper*. (See Supplementary Material 2: Working Paper).

With regard to philosophy, the creation of a nurturing, empowering, joyful environment was strongly encouraged. With regard to class content, the basics of mindful exercise and meditation and, to a lesser degree, integrated self-massage were endorsed. The statement that ‘healing comes from within’ was briefly discussed, then validated. With regards to instructor competence and knowledge, panel members voiced the opinion that Qigong including energy theory was the scope of practice, however some basic knowledge of cancer and its treatment were valuable. Several clinical considerations of importance were identified including movement or mobility restrictions, neuropathic paraesthesia, fatigue, pain, body image issues, impaired quality of sleep, and difficulty with concentration. Further, participant questions regarding medical management and concerns unrelated to qigong practice, nutrition or life style counseling, other than stress management specific to Qigong, were considered to be best referred to the most appropriate health professional. With regards to any discussion of current research, the majority of panel instructors (8/9) did address current research to one degree or another in their classes. However, a caution was proffered that instructors be careful not to misrepresent research findings, so as to offer false hope. With regards to confidentiality, panel members confirmed the importance of maintaining confidentiality of personal and health information volunteered by participants. 

## 4. Discussion

While preliminary, the information gained through this research has broad clinical implications. Foundational to recommendation generation is a belief that Qigong exercise in supportive cancer care programming has benefit and is complementary and integrative with, rather than alternative to, traditional medical management. All recommendations within the guidelines, serve to inform and guide rather than to regulate.

Primary content recommendations include practice of mindful exercise (without reference to style or form), meditation, and self-massage. There is a wide variation among the Qigong instructors and programs represented in terms of use of Qigong styles and forms. Structure of programs varies depending on school of thought and philosophy (e.g., daoist, yin and yang theory, energy-channel theory, and five phase (element) theory, emphasis on physical strength and prowess with martial art application, energy cultivation, or spiritual enlightenment with meditation or combinations therein). At least one instructor predominantly taught a traditional Tai chi form. Others reported teaching modified Tai chi forms combined with Qigong practice. While still others reported teaching only traditional or derivative Qigong forms. This variance was consistent with previous related research [4]. Similar to a 2010 expert panel working paper defining therapeutic Tai chi and Qigong [17], no attempt was made to compare or elevate one form or style as superior to any other.

Meditation has been recognized as an essential part of Qigong practice [18,19,20]. While there is no confirming clinical research specific to individuals with cancer, it may be that meditation has more clinical relevance for individuals with cancers and those who have chronic illness and their goals of practice as compared to others who practice Qigong exercise for general health, balance-training or neuro-motor retraining. Also, there was some panel discussion regarding inclusion of self-massage and as to what techniques constitute Qigong self-massage. For the purpose of delineation, self-massage was clarified within instructor competence guidelines as: 

Note: self-massage occurs naturally with exercise movements in the form of gentle stretching of the fascia, visceral massage, and gentle mechanical stimulation of articular surfaces within joints or may be applied as rubbing, tapping, body drumming or bone marrow washing, auricular massage, stroking of energy fields as in Reiki or therapeutic touch, even the vibration created from vocalization of healing sounds. [Source: Table 3]

While recommendations on specific frequency and duration of practice were not delineated in this work, panel members agreed that availability of instructional aids to facilitate home practice should be recommended. Research on the importance of consistency of practice is not available for individuals with cancer. Though some insight may be gained from research on dose-response for other clinical populations. Chan et al. in an RCT of 150 women with chronic fatigue syndrome found that the number of Baduanjin Qigong lessons attended and the amount of Qigong self-practice were significantly associated with sleep, fatigue, anxiety, and depressive symptom improvement [21]. Lynch et al. found similar results in post hoc analysis of Chaoyi Fanhuan Qigong exercise compliance in an RCT of 100 individuals with fibromyalgia [22]. Those who practiced per protocol (≥5 times/week) had significantly greater improvement than those who practiced minimally (≥3 times/week).

Panel members acknowledged the importance of instructor competence in Qigong exercise for cancer care. Participation in the research is considered tacit agreement that panel members invested in the belief that individuals with cancer are a vulnerable population and as such require instructor skills beyond entry level. These include competence within their craft, a general knowledge of cancer and associated adverse effects of cancer management, access to basic knowledge of current research in the area, as well as, sensitivities specific to the individual cancer journey. This knowledge expectation is beyond what might be expected of entry-level Qigong instructors where the student population is less vulnerable and health promotion is the primary goal of the Qigong practice. The guidelines for instructor competence, in terms of knowledge and skills for Qigong exercise in cancer care programming, can serve as a resource for among others, qigong instructors new to supportive cancer care, existing qigong instructors who value continuing professional development, educators who prepare this work force, and human resource professionals who staff programming.

### 4.1. Limitations

The current work has at least one major limitation related to validation. Validation is by consensus of expert opinion. Sampling method was one of convenience. By a priori design, the consensus panel sample does not include masters working in TCM settings. Voices of experts-in-the-field not included in the panel might have adjusted findings. Secondly, validity of recommendations is predicated on a belief that instructor competence directly influences outcome. 

### 4.2. Implications

This international Qigong expert panel recognizes that there is no one perfect standard Qigong program that is superior to any other. Nor can one program serve all individuals, nor integrate within all institutions. Nonetheless, panel members agreed common principles outlined in the consensus guidelines for structure and delivery of Qigong exercise in cancer care and recommendations regarding instructor competence generated from this research may serve as foundational resources to guide current and future Qigong exercise in cancer care programming. This initial work is the first step in bridging the gap between evidence-base and clinical practice. Panel work products can better inform individuals with cancer and the health professionals, educators, and policy-makers who serve them as to the structure of the Qigong exercise in cancer care programming as well as to provide stimulus for further collaborative discussion toward establishing best practice. In addition to afore stated implications, the current consensus guidelines may also serve as point of reference to facilitate communication among oncologist, patient and Qigong instructor when make a decision about integrative cancer care services and allocating healthcare resources. Finally, consumers of this research are encouraged to view the full *Qigong in Cancer Care Guidelines:* A Working Paper appended to this report (See Supplementary Materials 2: Working Paper), so that they may benefit from the insights gained by the pioneers who are forging practice in this important clinical area.

Future research, either collaborative or independent, is invited to replicate this work to include voices from other experts in the field for validation and comparative analysis. Additional future research may also assess level of effect in relation to competence of the instructors (e.g., novice vs. one with a measurable level of competence and number hours of experience). Prerequisite to this type of research is psychometric study, for example, development of standardized participant reported outcome tools to measure clinical outcome and a means to measure or certify level of instructor competence.

### 4.3. Adoption of Innovation

The value of any applied clinical research lies in how well it improves practice. Awareness of the existence of this work will be raised by submission of informational postings through the National Qigong Association, the Qigong Institute, the Qigong Network, various instructor training workshops including a post conference workshop of the International Society for Integrative Oncology November 2017, Chicago, IL, USA, and invitation for social sharing among professionals in the field. It is hoped that instructors in existing programs will assess and modify their programs based on recommendations generated by the panel. For those just entering this clinical area of practice, regardless of the Qigong style of preference, and for administrators planning new programming consideration of these recommendations can aid and guide structure of respective programming.

## 5. Conclusions

In conclusion, panel members agree that Qigong in cancer care be integrative with modern medical management. The environment should be nurturing, empowering and safe. The population served has special needs as compared to the general population, therefore the instructor should have a basic knowledge of cancer, its management and side effects of management to assure the safety of participants. Regardless of the style or form of Qigong taught in classes, the meditative and energy cultivation components of the exercises should be emphasized.

## Figures and Tables

**Table 1:** Summary of class time utilization for existing programming (*N* = 9 programs)

Setting	Health Care or Cancer Center 8		Community Setting 1	
Class length	45−60 min	60−90 min	3 h	
	6	2	1	
Time utilization by %:	Mean	Range	SD	
dynamic exercise	61.13%	30−75%	14.24	
meditation	15.23%	0−33.3%	10.29	
self-massage	12.29%	2−40%	11.03	
Is time dedicated to discuss healthy life style?	Yes		No	
	5		4	
Is time dedicated for peer support?	Yes		No	
	7		2	
How often do you discuss research?	Always	Sometimes	Rarely	Never
	1	5	2	1

**Table 2:** Consensus Guidelines for structure and delivery of Qigong exercise for cancer care programming.

*Program Philosophy* Healing comes from within. One has the innate power to improve health and healing through thought and lifestyle choices including regular practice of mindful Qigong exercise. *The primary goal of Qigong exercise for cancer care is to facilitate preservation and enhancement of quality of life associated with the cancer experience.* Qigong exercise classes are offered to serve the whole person as complementary to traditional medical management. Class activities involve gentle, mindful exercise including traditional qigong and/or therapeutic Tai chi, meditation and self-massage. Qigong instructor competence is integral to maximum achievement of program goals. Class environment is safe and one of empowerment, nurturing, safety, and joyfulness.
1. Program Administration 1.1. Select a qualified Qigong instructor to administer classes. 1.2. Reserve a quiet, calming class location. (Consider wheelchair accessibility and room size requirements.) 1.3. Request intake documentation on name, contact information, and emergency contact. 1.4. Employ a mechanism for assessing activity tolerance, special needs or safety concerns prior to class entry. This may be accomplished by interview or completion of a short screening form. 1.5. Use of a signed waiver for liability release for the facility and the instructor. 1.6. Provide for participant feedback and class evaluation. 1.7. Provide accommodation for mobility and activity tolerances. (e.g., provide chairs for sitting and possibly portable cots or yoga mats for lying down, based on class activities and participant needs). 1.8. Have a plan for emergency response and medical assistance.
2. Program Content 2.1. Instruction in a relatively easy-to-learn Qigong/Tai chi form, one that results in immediate benefit, and one that can be modified for a range of activity tolerance challenges as well as progression of learning. 2.2. Dedicated practice of mindfulness and meditation. 2.3. Inclusion of self-massage techniques as used in Traditional Chinese Medicine 2.4. Instruction for home practice of exercises as well as how to use techniques to reduce stress and anxiety in daily life. Provision of instructions for home practice in video or written format is recommended. 2.5. Schedule class length (approximately 60 min) to provide for a short period of social peer support before and after the actual class and sufficient rest time, as needed. Class time use should be fluid and responsive to participant needs. (A sample format is 10 min for assembly and group discussion; 5−10 min opening meditation and warm-up exercise; 40 min combination of active exercise, meditation, and self-massage; and 5−10 min closing discussion. Closing interactive discussion might include a review of any key points from the day’s lesson, a question and answer opportunity, information on energy theory, tips for home practice and coping strategies, optional updates on research, and/or Qigong experience sharing. Note: additional opportunities for class interaction may occur strategically during the class.)

**Table 3:** Consensus Guidelines for instructor competence in teaching Qigong exercise for cancer care classes.

1. Knowledge Domain 1.1. Knowledge of energy theory and energy cultivation. (Suggested concepts include 5 element theory, major meridian paths and energy gates, microcosmic and macrocosmic orbits, key acupressure points for addressing nausea and headaches.) 1.2. Knowledge and ability to teach program content: mindful exercise forms, meditation, and self-massage. (Note: self-massage occurs naturally with exercise movements in the form of gentle stretching of the fascia, visceral massage, and gentle mechanical stimulation of articular surfaces within joints or may be applied as rubbing, tapping, body drumming or bone marrow washing, auricular massage, stroking of energy fields as in Reiki or therapeutic touch, even the vibration created from vocalization of healing sounds.) 1.3. Skills of effective class management 1.4. Some basic knowledge of anatomy (major joints: spine, hip, knee, shoulder and locations of major organs, as would be expected for teaching any general health and wellness Qigong/Tai chi classes). 1.5. A very basic knowledge of what is cancer and treatment of cancer in both Western medicine and Chinese medicine theory. 1.6. Qigong can help to improve cancer-related quality of life, boost the immune system and regulate the inflammatory response. 1.7. Knowledge of any potential harmful effects of Qigong practice for individuals with cancer and, in particular, any special considerations or activity restrictions for class participants. 1.8. Ability to communicate with the public and other health professionals regarding (a) what constitutes Qigong in cancer care specific to your class activities, and (b) to advise participants to seek consul from other health care professionals for questions beyond your scope of practice or professional knowledge. 1.9. Knowledge of current research on Qigong as supportive cancer care is encouraged. (suggested Internet free-access resources: The Qigong Network: http://theqigongnetwork.com, the National Qigong Association webpage http://nqa.org and PubMed online research database.)
2. Psychomotor Domain 2.1. Instructor competence in the performance of selected Qigong style(s) or form(s). 2.2. Ability to modify exercises for varying levels of activity tolerance and any movement restrictions or limitations. 2.3. Training in basic life support is encouraged.
3. Affective Domain (demonstrate behaviors consistent with the following) 3.1. Maintaining the safety, dignity, and privacy of participants. (Note: one should adhere to strict privacy regarding any health information volunteered by participants as well as contact information.) 3.2. Teaching without ego. 3.3. Having strong empathy and a willingness to learn from the clients served. 3.4. Having a passion for the discipline and dedication to service.

## Supplementary Materials

The following are available online at www.mdpi.com/2305-6320/4/3/54/s1, Supplementary Material 1: Data, Supplementary Material 2: Working Paper.
