# Supplementary material for: International Expert Panel Consensus Guidelines for Structure and Delivery of Qigong Exercise for Cancer Care Programming
Source: Medicines (Basel). 2017 Jul 14;4(3):54. doi: 10.3390/medicines4030054 (PMC5622389; doi:10.3390/medicines4030054)
Supplement: Supplementary file 1 [file medicines-04-00054-s001.pdf]

Supplementary Materials: International Expert Panel Consensus Guidelines for Structure and Delivery of Qigong Exercise for Cancer Care Programming

Penelope Klein, George Picard, Roger Schneider and Byeongsang Oh

Supplementary Material 1: Data

The vast majority of guideline statements proposed in Round 1 were fully endorsed. However within Rounds 1 and 2, panel members offered several comments suggesting wording changes, emphasis placement, and additions. The following presents those comments categorized by theme and subtheme. Note: some comment statements are minority opinions, not adopted into the final Working Paper (Supplement 2.).

1. Format, Philosophy and Class Structure

1.1. Format

Keep it simple
Perhaps break guidelines into two sections: structure and Instructor competence.
I like the pithy statements

1.2. Philosophy

1.2.1. Regarding "Healing comes from within"

Essential to state "healing comes from within".

I would prefer this be more specific. Perhaps your intention was to emphasize the individual's contribution to healing. If so, we still may want to recognize it as being collaborative rather than an individual effort. Healing is complex, yet it is not possible without the individual's efforts to improve their outlook, their lifestyle and their approach to their well being. Healing requires the individual's physical emotional and spiritual participation in their quest for wellness (or wholeness or well being).

"Healing comes from within" – Many cancer patients feel they have done something to bring on their own cancer – maybe they have but a qigong teacher does not need to do anything to reinforce this notion, ie if healing comes from within then so does sickness. Might be changes to something along the lines of "Your thoughts can help you with your treatments and healing".

Many cancer patients feel they have done something to bring on their own cancer – maybe they have but a qigong teacher does not need to do anything to reinforce this notion, ie if healing comes from within then so does sickness. Might be changes to something along the lines of "Your thoughts can help you with your treatments and healing".

I think the comments on "healing comes from within" are valid and, obviously, "healing from within" does not apply to most cancer treatments. In my classes, we talk about tai chi as an on-going practice and as a tool for helping oneself. Toward that end, I often assign "homework" which can be a very simple movement. Not everyone practices between classes but some do and report that it helps them relax.

"Healing comes from within" yes it does. This should not be cause for blame or guilt on behalf of the student. It is good news, delivered correctly should empower. If a student were to construe this as a cause for blame, "Many cancer patients feel they have done something to bring on their own cancer" this is an opportunity for the teacher to lead the student away from this sort of non-constructive thinking. To take responsibility that yes, the lifestyle choices made in the past have a large part to play in the students current situations, but the other side of that truth, is that the student can now take 100% charge of the future situation. We need to address the issue with courage, not fear. Telling the students, "Your thoughts can help with your treatments and healing "is not giving a clear

message. Your thoughts affect everything, everyday for good and bad. Shielding them from that truth is not constructive.

Re: "Healing comes from within", it does.

1.2.2. Regarding empowerment

I support the collaboration. It takes a village and while we want to bring best our in all of us, we also want guidance and support.

I prefer to introduce myself to students as more facilitator than teacher. I firmly believe that not only all healing, but all learning comes from within, so in my class the student would be introduced to their internal teacher by tapping into the non judgmental observer within. This leads the student towards deep understanding of themselves but also a sense of responsibility. Even though the teacher manages the class, looks after all aspects of the energy in the room and the well being of each student, the student is actively looking after themselves. I am mentioning this here because I feel there is a common thread running through this review. I get the sense that, if we over complicate our remit as teachers, we can disempower our students. Every individual who walks through our doors is different in so many ways .I sincerely believe that each one has an ability to take responsibility for their learning, physical status and mental health. Over regulating our methodology and indeed the language we use as teachers can stunt the relationship between teacher and student and inhibit the flow of information.

We empower. Give control over symptoms.

In all class environments, each student has a different set of abilities and needs. It would be wise for the teacher to understand that they are there to facilitate and empower the student. This would lead the teacher to encourage the student to investigate and learn the various aspects of their personal illness, to take responsibility for their physical bodies. The student would be in control of how much effort/energy they could give to practice in any given day. The student/patient would also learn from their medical team the nuts and bolts of the cancer they are suffering from and take professional advice from the relevant professions. This should assist the student to take ownership of the situation. On the same hand, as a cancer patient and Tai Chi teacher, I can testify that a cancer diagnosis can shake one to the core and the sense of lack of control can be overwhelming. The tai chi class can assist in giving back some personal control and empowerment if the teacher does not overreach his/her remit.

We can build compassion and understanding through working as a team with different skills and talents. This also assists the students to accept where they are without judgment. The student then is required to take charge and know his/her limitations on any given day.

1.2.3. Re Inner Peace

Would like to see a bit more language that includes a sense of inner peace, a focus on being present in the body fully, in the moment, and with a sense of self-nurturing and the creative forces the energetic reception and cultivation of the many forces of nature.

I agree with {previous comment}: "Would like to see a bit more language that includes a sense of deep inner peace, a focus on being present in the body fully, in the moment, and with a sense of self-nurturing, a meditative state that includes a sense of connection with all of nature, and the creative forces".

1.2.4. Regarding atmosphere of the class

Having fun is very important too. Make the class atmosphere one where the participants feel safe and cared for.

I advocate for lightheartedness and a sense of humor.

Make it fun, if you want people to continue.

Fun is fine if its respectful.

I would advocate for a light heartedness and sense of humor. These traits in the teacher can be invaluable in keeping the students engaged and comfortable with their abilities.

Would like to see a bit more language that includes a sense of deep inner peace, a focus on being present in the body fully, in the moment, and with a sense of self-nurturing, a meditative state that includes a sense of connection with all of nature, and the creative forces (wording less scientific like "the energetic reception and cultivation of the many forces of nature").

1.2.5. Regarding class should be "supportive, empowering and fun"

Would refer to see 'support' as a stronger word, like ' nurturing', and 'fun' more like 'joy' This is where we can start overcomplicating our role. We need to be able to use language in a fluid manner to facilitate the particular students in our class. Language use is an important tool and should be versatile to suit cultural, age and various demographics.

When I interviewed for this teaching position the Exercise specialist told me that many people don't stick with support groups, they find them too depressing. As a result they came to exercise classes to focus on more positive things. With that in mind I always try to make it fun. I believe that it is essential that classes are a place where people can step away from their illness and find a supportive, uplifting atmosphere in which to spend a little time and to walk away from with a little peace of mind.

1.2.6. Regarding class should be "supportive, empowering and fun"

Make class atmosphere is one where patients feel safe and cared for.

Atmosphere should feel safe.

1.2.7. Regarding QOL

Tai chi can improve QOL for cancer patient.

Cancer care classes are intended to preserve and/or enhance QOL.

The majority of the cancer patients do get better and for me I think we need to not only preserve their quality of life but enhance it as well.

Participants should leave class feeling better than when they came in.

Understanding of Qi can improve medical symptoms, assist healing, and preserve energy, etc.

Some patient tell me that their tai chi is essential to their recovery.

Cancer Care Qigong classes are intended to preserve and/or enhance quality of life associated with the cancer experience. (I have my participants evaluate the program at the end of each 8 week session and all of them feel that their quality of life has been enhanced in some way.)

The majority of cancer patients do get better and for me I think we need to not only preserve their quality of life but enhance it as well.

1.2.8. Complementary to medical care

I really like the bullet that explains that Qigong is offered to serve the person as complementary to traditional medical management. How about saying: complementary to traditional medical treatment/management.

Definitely emphasize as complementary.

Agree – complementary

1.3. Structure

1.3.1. Regarding Location

Once health and safety levels have been adhered to, location should be in all aspects of normal day to day life. Creating a special environment, can impede the practitioners learning that their peacefulness is unconditional.

Make sure it is wheelchair accessible.

Agree – quiet and free from distractions.

1.3.2. Regarding enrollment composition

Have a mechanism for screening appropriateness for joining a group class.

Keeping all levels working in the same class will offer more benefit than dividing classes into subgroups in most cases. When dealing with a cancer group, you will still have a varying level of physical ability. This goalpost will change on a weekly basis as different students are at various stages of treatment. More can be learned by everyone if the class facilitates standing, sitting moving lots and not moving so much. All students will be all of these levels at some stage or another.

Consider offering classes specific to subgroups.

- New students;
- Sitting: individuals with activity restrictions or tolerance;
- Continuing students - then progress to recovered, more active, more advanced forms.

1.3.3. Regarding Class time use

Would add a potential for fluidity in class time use to allow for questions plus a change in direction guided by students needs/ circumstances.

Adjust to class.

1) I think that a class longer than 60 minutes in length is too physically taxing for most people. An 80 minute class is too long and too arduous for most people to commit to on a regular basis. I believe that the discussion (stress management, nutrition, lifestyle, etc.) portions of the class time use section would be better served (and expanded upon) in a separate class. Discussions of home practice can be easily integrated into class time. I am in my classroom 10 minutes before class begins so that people can gather and interact with each other and we can devote class time to Tai Chi/QiGong.

2) And to take breaks and sit when the need be.

1.3.4. Regarding class content:

"Practice dedicated to energy cultivation, mindfulness and meditation".

Would like to see a bit more language that includes a sense of deep inner peace, a focus on being present in the body fully, in the moment, and with a sense of self-nurturing, a meditative state that includes a sense of connection with all of nature, and the creative forces (wording less scientific like 'the energetic reception and cultivation of the many forces of nature') movement, breathing imagery NOT much discussion as it can raise stress levels.

Understanding of Qi.

Instruct students in how Qi flows and nourishes the body and ones healing. Describe 5 element theory in relation to organ function.

1.4. Content

1.4.1. Regarding forms/exercise

Exercise, meditation, self-massage essential.

Easy forms to begin with, but allow progression.

Along with easy-to-learn Qigong/Tai chi form. Somewhere state that some forms may progress based upon a participants' ability (my students that have been with me for more than one session like that I continue to challenge them in the area of strength, flexibility and balance). Also that Qigong can be done sitting in a chair and participants are encouraged to sit when needed. I always have my participants regardless of their fitness level have a chair in front of them.

Would include exercises for strength, flexibility and balance.

Allow sitting.

This could be looked at from the perspective of the client who, will benefit greatly to being challenged to assist mental agility following treatment/confinement.

We have some students who have been taking this class for several years who enjoy and are motivated by some complexity. Also, what is easy for some may be very difficult for others.

It is a challenge to shape a class to meet the needs of all but that should be a priority.

EASY – this could be looked at from the perspective of the client who, will benefit greatly to being challenged to assist mental agility following treatment/confinement.

1.4.2. Regarding class content: breath regulation

Regulation too strong a word – try breath awareness.

I do not teach breath regulation – 'just breathe naturally'.

I think its ok to teach this and to learn to coordinate with movement, BUT there needs to be heightened awareness that controlling breath can have many many side effects, and in a group in which some can move slow and breathe slow and other can not, there is great risk and forcing unsafe pace for some trying to match rest of group.

Regulation seems like a fine word here. We can be aware of our breath and not be breathing correctly or enough. Regulation, suggests we are actively taking charge of our breath, regulating the pace and tempo, coordinating our movements to it while having awareness of it.

Adjusting breathing to 'opening and closing' movements is taught in our classes.

I teach abdominal breathing.

1.4.3. Regarding class content: mindfulness/meditation:

I do emphasize 'mindfulness'. I not teach meditation.

Should be Knowledge and ability to teach meditation and mindfulness (I strongly agree that this is important) Teaching your students to be present and not dwelling on the past which though we have great memories is normally associated with grief and sadness. And also not to dwell on the future which is normally a wanting for things to be different or a fear of what may happen. Teaching them to be present is so calming.

Meditation is the 'Golden Treasure'

Meditation is essential, especially for this population

Tai Chi/Qigong is moving/standing meditation. The word mindfulness is relatively new. I wouldn't rule ether out.

1.4.4. Regarding class content: self-massage

Self-massage should include acupressure points for nausea and headache, among others.

I do not use much self-massage.

Self-massage this many be appropriate for some but not all students. Some will be medically advised no to use it.

Some patient might be instructed (by physician) not to use self-massage.

Many of my students feel uncomfortable with many self-massage techniques. My students tend to be older and more conservative (we are in the Bible belt). These techniques must be chosen carefully according to the population served. Additionally, rubbing, body drumming & tapping can be difficult for students that are post surgery or undergoing radiation therapy and may be experiencing skin burns or sensitivity.

Massage can be light, like laying of hands—self reiki, budhha plam, etc.

1.4.5. Regarding class content: Life style/nutritional advice

Depends on knowledge of instructor not primary to learning Qigong, leave to the professionals if not qualified.

I really like the fact that the draft mentions that Qigong is complemented by healthy nutrition and how one perceived and responds to life experiences.

Depends on knowledge of instructor and context. Must be sensitive to patients/students already being told what to do and not do by so many others. Sometimes good to simply guide in tai chi/qigong and preach less.

Unless the instructor is a certified therapist, I don’t think we are qualified to talk about stress management as to how we perceive & to respond to life events, except in the most general of terms.

1) I do think that it is imperative that the instructor model such behavior, but not to necessarily be the “expert” on nutrition or stress management.

2) When it comes to lifestyle lessons, the lessons we learn through tai chi practice and philosophy are not designed solely for tai chi time. The idea is that we integrate these lessons into our day to day lives. With this in mind, reference to lifestyle would always factor in any of my classes. On a personal note, I have found wisdom and solas from the teaching, while dealing with the initial shock and the following dramatic changes that happened in my life over the months. Tai Chi helped me to stay grounded to understand change and how to process what was happening.

3) I am neither a therapist nor a nutritionist and don’t feel it is appropriate to advise in areas outside of my field.

4) Unless the instructor is a certified therapist, I don’t think we are qualified to talk about stress management as to how we perceive & respond to life events except in the most general terms

1.4.6. Regarding class content: home practice

Provide DVD or manual.

Some report they use breathing and calming exercises when confronted with stressful situations would add journaling.

It is difficult to get people to practice on their own, I always provide a handout.

DVD’s or manual instructions needed for home practice.

Add some instructions for home practice e.g. don’t practice on a full or empty stomach, avoid cold food or drinks after practice, avoid excessive smoking or sex over the period of qigong practice

Other:

Encourage drinking of water to flush toxins.

Also add knowledge of the 3 Hearts (The first heart is the heart in their chest, Lower Abdominal breathing is considered the second heart, and the third heart is their calf muscle).

Would add ability to observe emotions and cultivate emotional maturity. Also assisting the practitioner to build awareness of mind/body connection.

Would add journaling. This would exponentially assist practitioners learning experience, particularly those undergoing chemotherapy as there are memory issues.

Classes should be free

[re: Free classes] This should be encouraged when possible, but not enforced.

Another aspect of our classes that might interest you is that our classes are offered for the care givers of cancer patients as well as the patients themselves. We have spouses and children of patients in class, normally with the patient but sometimes by themselves. They come for support and to help manage the stress of their circumstance.

Our classes include family or friends for support and are offered for free.

I think tai chi is especially helpful for balance.

Consistency of daily practice is very important.

It is not how much knowledge of qigong that is important. One must practice daily.

1.5. Instructor competence

1.5.1. Regarding knowledge of Integrative Oncology

I do agree that it is an important component to overall integrative therapy, but is best served in a setting other than that of a Tai Chi/QiGong class.

I think it is beneficial, but not essential, for instructors to have knowledge of Integrative Oncology methods.

Think it is important for an instructor to know: integrative oncology {approach to cancer management}, energy cultivation, mindfulness, self-massage, energy gates.

What if they have some medical background, e.g. acupuncturist or PT, but not specifically integr onc. I think this would be ok.

1.5.2. Regarding instructor knowledge of cancer

The cancer journey does not look the same for everybody, even if they have the same type of cancer.

Cancer is a collection of diseases manifesting as tumors. There is wide variation among them.

Instructor should know something of cancer.

Basic knowledge would be good.

I agree that a basic-level knowledge of the genesis, treatment & management of cancer is very beneficial. As a suggestion, I believe that instructors who have not been personally touched by cancer would find great insight by touring an oncology department or cancer center and speaking with an oncology nurse.

It is not an instructors place to take any position, for or against, any treatment or modality. We are not physicians, nor do we have access to the students medical records and are, therefore, completely unqualified to offer any opinion. Any discussion of treatment or modalities should be immediately referred back to the attending physician.

1.5.3. Regarding instructor knowledge of research

Helpful, but important not to misrepresent.

I agree that it is important to have knowledge of current research on Tai Chi/QiGong as regards to all aspects of health, not just cancer care. However, it is imperative that instructors do not create false hope or false expectations for cancer patients/survivors. The presentation of research findings should be handled with care. Patients / survivors are in a vulnerable state of mind and can easily mis-interpret findings.

1.5.4. Regarding instructor motor skill

One should be expert in their craft.

Should be able to modify for exercise restriction or limitations.

Instructor should be able to adjust and modify their program for some individual who has impaired physical movement during the medical treatment time to time.

Add CPR.

CPR should be mandatory.

1.5.5. Regarding clinical considerations

Be familiar with the terms: lymphedema, paraesthesia, placebo, chemo brain, fatigue issues, memory loss, concerns over hair loss, diabetes, difficulty concentrating.

Let participants know we all have challenges and some days we feel better than others and that is normal; work within comfortable range, rest when needed. Have fun.

I think having experience teaching Qigong to participants with age associated diseased besides cancer is a plus such as cardiovascular disease, arthritis, diabetes, Parkinson's, hypertension, COPD, vision and Hearing loss, and osteoporosis.

I think we should add to the major joints: ankles, elbows, and wrists. To the major organs add gall bladder, spleen, stomach and pancreas. (to knowledge)

I would refer them to their doctor if they told me. Most of my participants don't talk to me about their symptoms.

How Qigong can improve medical symptoms, assist healing, prevent disease, and preserve energy etc? (surgery, chemotherapy, radiotherapy and hormone theapy as well as healthy lifestyle)

Mind body/intention, connection work, will benefit students suffering with parenthesis. There would be a strong case to spend considerable time on this aspect of Qigong.

Understanding of Qi can improve medical symptoms, assist healing, and preserve energy, etc.

I think having experience teaching Qigong to participants with age associated diseases besides cancer is a plus such as cardiovascular disease, arthritis, diabetes, Parkinson's hypertension, COPD, vision and Hearing oss,and osteoporosis.

Memory loss, difficulty concentrating, fatigue, concern over personal appearance, difficulty balancing, in addition some patients have other health problems: diabetes, arthritis, weight issues.

"Chemo Brain", Range of Motion, Paresthesias or Neuropathy, Shortness of Breath, Fatigue, Joint Pain, Skin Sensitivity and/or Burns from Radiation Therapy.

It is also important to be aware of student's discomfort if they have a "port" or other medical device.

1.5.6. Regarding Safety

A general announcement at the beginning of each class regarding "doing things within your limits" and if any movement hurts or feels uncomfortable to let the instructor know so that modifications can be suggested is very important. Any health issues should be referred back to their physician.

Need to know what you don't know and when to send to cancer professional, if problems arise.

We have a pre-registration form for everyone to complete which touches on physical limitations but which is not as thorough as what you've suggested. I think the lightheadedness cautions are something I will definitely incorporate. I'm less sure about the recommendation for diabetics but will consider it further.

Also, that they are to work within their range of motion on any given day.

Tell any individuals with diabetes to eat something prior to class and to bring a ready sugar to class.

1.5.7. Regarding communication with other health professionals

I don't have any occasion to do so.

Refer participant to seek out advice from others more qualified: eg. for nutrition advice or feelings of doom or preoccupation with death.

Instructors should consider writing progress reports to oncologists at least twice a year.

Any discussion of treatment or modalities should be immediately referred back to attending physician

1.5.8. Regarding confidentiality of information

HIPA standards need to be observed at all times. If the class is being held at a medical facility, it is specifically forbidden to ask any student about their health or limitations. Most instructors are contract employees or volunteers, not staff and are not covered by the medical facility's access or right to such knowledge. It also becomes a liability risk if the instructor has been given specific knowledge about a student and an exercise causes them problems, even if they are doing it voluntarily.

Instructors cannot discuss participant medical information within the class, but participants, themselves, may share medical and personal information, if it is their wish to do so.

If one is working in a cancer care center, there are strict rules about confidentiality, these practices should be known, adhered to and carried into community settings as well.

2. General Advice from the field

Making sure to let the participants know that we all have challenges and some days we feel better than others and that is normal.

Don't preach; They are being told what to do by so many.

Ask yourself, why do you want to teach?

Listen. Don't Tell.

Don't limit exercise time by talking too much

Listen 2x's more than you speak.

Listen and learn from your students

People don't care how much you know until they know how much you care.

Several additional comments that could be categoried as 'Advice from the field' are imbedded in previous comments.

Our goal is to help patients move beyond cancer with wellness for life. It can be the most powerful medicine of all.

How about saying something about the cancer journey.

1) I would strongly suggest adding to program logistics, a recommendation of a personal liability insurance policy.

2) Instructors should consider carrying liability insurance if they do not already do so.

3) An emergency contact should be a part of any waiver for liability release and not part of intake documentation

The cancer journey is an individual experience, even if they have the same type of cancer.

Supplementary Material 2: Working Paper. Working Paper on Consensus International Expert Panel Guidelines: For Structure and Delivery of Qigong Exercise for Cancer Care Programming

Jan 20, 2017

Convening members: [to be inserted following review]

Acknowledgement: The convening panel member/authors wish to acknowledge and thank the additional expert panel members who assisted in this research. They include: Cheryl Roland Weston, White Jade Tai chi, Greensboro, NC; Linda Larkey, Ph.D. College of Nursing and Health Innovation, Arizona State University; Nancy L. Mayhem, Wellness Coordinator, Cedar Crest, Inc, Janesville, WI; Nadine Buttery, Tai chi and Qigong instructor; Todd Binzen, Qigong instructor; Gerald Hopkins, MAW, University of Iowa; Peter M. Wayne, Ph.D.; Associate Professor of Medicine, Harvard Medical School, Director of Research, Osher Center for Integrative Medicine; and Ramel Rones, Qi Gong and Tai Chi Master, Boston MA.

Introduction:

Qigong exercise has been an integral component of traditional Chinese medicine for millennia. Recent scientific research has validated that among its many therapeutic applications that Qigong exercise can improve cancer-related quality of life. Theoretical research suggests that it may also have a supportive role in cancer prevention and improved survival.

There are an estimated 15 million cancer survivors in the US. Approximately 15 million new cases are diagnosed annual through out the world. In China, Qigong exercise is widely used in management of cancer, however, little is known of this ancient health-promoting modality in the West. In our global world, as we become more familiar with Qigong exercises and its therapeutic potential, there is an anticipated unmet service need for Qigong programming to serve individuals with cancer.

In response to this perceived need, a panel of international Qigong instructors with knowledge of serving individuals with cancer was convened from Nov 2016 through Feb 2017. The vision of this work was to advance acceptance of Qigong exercise as a therapeutic modality within Integrative Oncology. The specific purpose of the panel work was to develop guidelines to assist in developing and delivering quality Qigong exercise for cancer care programming. The resultant resources generated as panel products are detailed in the following.

Consensus Expert Panel Products:

Expert Panel work resulted in the generation of 2 guidelines, 2 tools, and a commentary from the experts in the form of a series of statements entitled 'Advice from the field'.

These include:

1) Consensus Guidelines for structure and delivery of Qigong exercise for Cancer care programming,

2) Consensus guidelines for establishing instructor competence for teaching Qigong exercise for cancer care classes,

3) Screening tool for safe participation in Qigong exercise,

4) Class participant instructions for maintaining safety during Qigong exercise, and

5) 'Advice from the field'.

Consensus Guidelines for structure and delivery of Qigong exercise for cancer care programming

Program Philosophy
• Healing comes from within.

- One has the innate power to improve health and healing through thought and lifestyle choices including regular practice of mindful Qigong exercise.
- The primary goal of Qigong exercise for cancer care is to facilitate preservation and enhancement of quality of life associated with the cancer experience.
- Qigong exercise classes are offered to serve the whole person as complementary to traditional medical management.
- Class activities involve gentle, mindful exercise including traditional qigong and/or therapeutic tai chi, meditation and self-massage.
- Qigong instructor competence is integral to maximum achievement of program goals.
- Class environment is safe and one of empowerment, nurturing, safety, and joyfulness.

1. Program Administration

1.1. Select a qualified Qigong instructor to administer classes.
1.2. Reserve a quiet, calming class location. (Consider wheelchair accessibility and room size requirements.)
1.3. Request intake documentation on name, contact information, and emergency contact.
1.4. Employ a mechanism for assessing activity tolerance, special needs or safety concerns prior to class entry. This may be accomplished by interview or completion of a short screening form.
1.5. Use of a signed waiver for liability release for the facility and the instructor.
1.6. Provide for participant feedback and class evaluation.
1.7. Provide accommodation for mobility and activity tolerances. (Eg. provide chairs for sitting and possibly portable cots or yoga mats for lying down, based on class activities and participant needs).
1.8. Have a plan for emergency response and medical assistance.

2. Program Content

2.1 Instruction in a relatively easy-to-learn Qigong/Tai chi form, one that results in immediate benefit, and one that can be modified for a range of activity tolerance challenges as well as progression of learning.
2.2 Dedicated practice of mindfulness and meditation.
2.3 Inclusion of self-massage techniques as used in Traditional Chinese Medicine
2.4 Instruction for home practice of exercises as well as how to use techniques to reduce stress and anxiety in daily life. Provision of instructions for home practice in video or written format is recommended.
2.5 Schedule class length (approximately 60 min) to provide for a short period of social peer support before and after the actual class and sufficient rest time, as needed. Class time use should be fluid and responsive to participant needs. (A sample format is 10 minutes for assembly and group discussion; 5-10 min opening meditation and warm-up exercise; 40 min combination of active exercise, meditation, and self-massage; and 5-10 min closing discussion. Closing interactive discussion might include a review of any key points from the day’s

lesson, a question and answer opportunity, information on energy theory, tips for home practice and coping strategies, optional updates on research, and/or Qigong experience sharing. Note: additional opportunities for class interaction may occur strategically during the class.)

Consensus Guidelines for instructor competence in teaching Qigong exercise for cancer care classes

1. Knowledge Domain
    1.1. Knowledge of energy theory and energy cultivation. (Suggested concepts include 5 element theory, major meridian paths and energy gates, microcosmic and macrocosmic orbits, key acupressure points for addressing nausea and headaches.)
    1.2. Knowledge and ability to teach program content: mindful exercise forms, meditation, and self-massage. (Note: self-massage occurs naturally with exercise movements in the form of gentle stretching of the fascia, visceral massage, and gentle mechanical stimulation of articular surfaces within joints or may be applied as rubbing, tapping, body drumming or bone marrow washing, auricular massage, stroking of energy fields as in Reiki or therapeutic touch, even the vibration created from vocalization of healing sounds.)
    1.3. Skills of effective class management
    1.4. Some basic knowledge of anatomy (major joints: spine, hip, knee, shoulder and locations of major organs, as would be expected for teaching any general health and wellness Qigong/tai chi classes).
    1.5. A very basic knowledge of what is cancer and treatment of cancer in both Western medicine and Chinese medicine theory.
    1.6. Qigong can help to improve cancer-related quality of life, boost the immune system and mediate the inflammatory response.
    1.7. Knowledge of any potential harmful effects of Qigong practice for individuals with cancer and, in particular, any special considerations or activity restrictions for class participants.
    1.8. Ability to communicate with the public and other health professionals regarding (a) what constitutes Qigong in cancer care specific to your class activities, and (b) to advise participants to seek consul from other health care professionals for questions beyond your scope of practice or professional knowledge.
    1.9. Knowledge of current research on Qigong as supportive cancer care is encouraged. (suggested Internet free-access resources: The Qigong Network: http://theqigongnetwork.com, the National Qigong Association webpage http://nqa.org and PubMed online research database.)
2. Psychomotor Domain
    2.1. Instructor competence in the performance of selected Qigong style(s) or form(s).

2.2. Ability to modify exercises for varying levels of activity tolerance and any movement restrictions or limitations.
2.3. Training in CPR is encouraged.

3. Affective Domain (demonstrate behaviors consistent with the following)
   3.1 Maintaining the safety, dignity, and privacy of participants. (Note: one should adhere to strict privacy regarding any health information volunteered by participants as well as contact information.)
   3.2 Teaching without ego.
   3.3 Having strong empathy and a willingness to learn from the clients served.
   3.4 Having a passion for the discipline and dedication to service.

Screening tool for safe participation in Qigong exercise

In order to maintain your safety while participating in Qigong Exercise classes, please complete the following brief screening survey. If you answered **Yes** to any of the questions below, then discuss same with Qigong instructor prior to joining the class.

- Are you limited in your ability to engage in mild exercise (equivalent to putting away groceries)?
- Has your doctor or health care professional advised you to limit or avoid specific physical movements or activities?
- Do you lose your balance at times or experience dizziness or lightheadedness when bending over to pick something up off the floor or when rising from a chair?
- Are you limited in your cognitive ability to maintaining your own safety during exercise?

Class participant instructions for maintaining safety during Qigong exercise

Please be aware that you, as a class participant, have a role in maintaining your safety during exercise participation.

- Discuss any exercise concerns with the class instructor.
- Wear comfortable, loose-fitting clothes and flat-soled, non-slip shoes to class.
- Bring a container of water to flush toxins and for rehydration during class.
- Only exercise within a comfortable range.
- Choose to sit, if you become fatigued or balance is a problem during exercise.
- Limit movement such as reaching up or bending down, if advised to do so by your health professional.

    - Limit activity such as self-massage, if it results in discomfort or you have been advised to do so by your health professional.
    - If diabetic or experience periods of low blood sugar, be sure to eat something prior to class and bring a ready sugar substance to take during class, for use if needed.

Tips for safe and effective home practice:

- For home practice, follow the guidance of your class instructor.
- Strive to practice daily
- Calm the mind and relax prior to starting your Qigong practice.
- Do not practice in an overly cold or hot environment (e.g. in front of air conditioning or outside when it's cold and windy or very hot)
- Do not consume cold drinks or frozen foods, such as ice cream, immediately before or after practicing Qigong (a warm herbal tea or room temperature drink is recommended).
- Try to practice at least one hour after a meal (e.g., lunch or dinner), it is not good to practice when one is feeling hungry or full.
- Avoid practicing after consuming alcohol.
- If any exercise results in pain or discomfort, stop the exercise and discuss with your instructor or your medical consultant before continuing.

Advice from the field

- Know your craft well.
- Nurture, encourage, openly express the joy of Qigong practice in order to guide students to self-discover the joy in their own practice.
- Keep it light and fun.
- Allow your students the experience. Talk less. Listen more. You will teach your students how to practice Qigong, and they teach you to become a better teacher.
- Only advise within your area(s) of expertise. Qigong is the focus, not medical treatment of cancer or nutritional counseling.
- Prepare and distribute a brochure or informational handout describing your program, its philosophy and activities that your students can share with their attending oncologist.
- Explore opportunities to defray participation cost including third-party reimbursement as health and wellness programming.
- Accurately interpret the research. There is strong evidence that Qigong practice can improve cancer-related quality of life. At this time (Jan 2017), research evidence suggests that Qigong practice may have a role in cancer prevention and improved survival, but definitive proof of these two potential benefits needs to be assessed and verified through additional population research.
- Each participant has a unique cancer journey. Perhaps all you need to know of cancer is that it is a collection of diseases most often manifesting as tumors.

Western management may involve surgery, chemotherapy, radiation, and/or use of medication. Integrative oncology adds complementary therapies. Traditional Chinese Medicine includes qigong practice and nutritional support.

- Some students may be experiencing paraesthesia (pins and needles sensation in feet and/or hands), difficulty concentrating, pain, fatigue, nausea, sleep disturbance, and emotional distress as side effects of the cancer and its treatment. These symptoms may persist during and after treatment. Qigong can help.
- Be aware that some of your students may be adjusting to physical changes due to surgery (e.g. mastectomy, colostomy) or hair loss from treatment. Guide them to see the beauty in their energetic selves. While the physical may be limited and less than perfect, the spirit can still abound.
- Consider it normal that some days are better than others for your students. For many, every time they come to class is a reason for celebration. Celebrate with them.
- Finally, your students won't care how much you know, until they know how much you care.

Additional Resources

The following are offered as informational resources that may be helpful for developing and marketing of new 'Qigong Exercise for Cancer Care Programming'.

For a general overview of Qigong exercise for Cancer care, see:

1 Klein, P. Qigong in cancer Care: theory, evidence-base, and practice. *Medicines* **2017**, *4*, doi:10.3390/medicines4010002.

For published evidence-base references supporting utilization of Qigong exercise for cancer care, see:

1. Jemal, A.; Center, M.M.; DeSantis, C.; Ward, E.M. Global patterns of cancer incidence and mortality rates and trends. *Cancer Epidemiology Biomark. Prev.* **2010**, *19*, 1893–1907.
2. American Cancer Society. Cancer Facts & Figures 2017. Available online: https://www.cancer.org/research/cancer-facts-statistics/all-cancer-facts-figures/cancer-facts-figures-2017.html (accessed on 14 March 2017).
3. Lui, T.; Qiang, X.M.; Eds. *Chinese Medical Qigong*; Singing Dragon/Jessica Kingsley Publishers: London, UK, 2010, ISBN: 978-0-85701-017-9.
4. Klein, P.J.; Schneider, R.; Rhoads, C.J. Qigong in cancer care: A systematic review and construct analysis of Qigong therapy. *Support. Care Cancer*. **2016**, *24*, 3209–3222, doi:10.1007/s00520-016-3201-7.
5. Lui, P.; You, J.; Loo, W.T.Y.; Sun, Y.; He, Y.; Sit, H.; Jia, L.; Wong, M.; Xia Z, Zheng, X.; Wang, Z,; et al. The efficacy of Guolin-Qigong on the body-mind health of Chinese women with breast cancer: a randomized controlled trial. *Qual. Life Res.* **2017**, doi:10.1007/s11136-017-1576-7.
6. Ho, R.T.H.; Wang, C.W.; Ng, S.M.; Ho, A.H.Y.; Ziea, E.T.C.; Wong, V.T.; Chan, C.L.W. The effect of t'ai chi exercise on immunity and infections: a systematic review of controlled trials. *J. Altern. Complement. Med.* **2013** *19*, 389-396.
7. Irwin, M.R.; Olmstead, R.; Breen, E.C.; Witiarama, T.; Camilo, C.; Sadeghi, N.; Arevalo, J.M.G.; Ma, J.; Nicassio, P.; *et al*. Tai chi, cellular inflammation, and transcriptome dynamics in breast cancer survivors with insomnia. *J. Natl. Cancer Inst. Monogr*. **2014**, *2014*, 295–301.
8. Yang, Y.; Verkuilen, J.; Rosengren, K.S.; Mariani, R.A.; Reed, M.; Grubisich, S.A.; Woods, J.A. Effects of a Taiji and Qigong intervention on the antibody response to influenza vaccine in older adults. *Am. J. Chin. Med.* **2007**, *35*, 597-607.

9. Wayne P.M.; Berkowitz D.L.; Litrownik, D.E.; Buring, J.E.; Yeh, G.Y. What do we really know about the safety of tai chi? A systematic review of adverse event reports in randomized trials. *Arch. Phys. Med. Rehabil.* **2014**, *95*, 2470-2483.
10. Li, F.; Harmer, P. Economic evaluation of a Tai JI Quan intervention to reduce falls in people with Parkinson disease, Oregon, 2008-2011. *Pre. Chronic. Dis.* **2015**, doi:10.5888/pcd12.140413.
11. White, J.D.; Lin, H.; Jai, L.; Wu, R.S.; Lam, S.; Li, J.; Dou, J.; Kumar, N.; Lin, L.; Lao, L. Proceedings of the Strategic Meeting for the development of an International Consortium for Chinese Medicine and Cancer. *J. Glob. Oncol.* **2016**, doi:10.1200/JGO.2016.005710.
12. Greenlee, H.; Balneaves, L.G.; Carlson, L.E.; Cohen, M.; Deng, G.; Hershman, D.; Mumber, M.; Perlmutter, J.; Seely, D.; Sen, A; et al. Clinical Practice Guidelines on the Use of Integrative Therapies as Supportive Care in Patients Treated for Breast Cancer. *Natl. Cancer Inst. Monogr.* **2014**, *50*, 346–358, doi:10.1093/jncimonographs/lgu041.
13. NIH Consensus Development Panel on Acupuncture-Acupuncture. *JAMA.* **1998**; *280*, 1518-1524.
14. Filshie, J.; Hester, J. Guidelines for providing acupuncture treatment for cancer patients--A peer-reviewed sample policy document. *Acupunct. Med*. **2006**, *24*, 72–82.
15. Chodzko-Zajko, W.; Jahnke, R. *National Expert Meeting on Qigong and Tai Chi: Consensus Report*; University of Illinois at Urbana-Champaign: Urbana, IL, USA, 2005. Available online: http://healerwithinfoundation.org/National_Expert_Meeting/ (accessed on 10 October 2016).
16. Klein, P. Qigong in cancer care: theory, evidence-Bbase, and practice. *Medicines* **2017**, *4*, doi:10.3390/medicines4010002.
17. Yang, M.Y. The Treasure Missing in Many Tai Chi and Qigong Practices. Available online: http://www.americantaichi.net/TaiChiKungLivingArticle.asp?cID=3&sID=6&article=TreasureMissing&subject=daily%20life (accessed on 21 February 2017).
18. Chen, C.W. Introduction to Chinese Taiji Five-Element Qigong. Available online: http://yang-sheng.com/?p=11022 (accessed on 14 March 2017).
19. Campo, R.A.; Light, K.C.; O'Conner, K.; Nakamura, Y.; Lipschitz, D.; LaStayo, P.C.; Pappas, L.M.; Boucher, K.M.; Irwin, M.R.; Hill, H.R; *et al*. Blood pressure, salivary cortisol, and inflammatory cytokine outcomes in senior female cancer survivors enrolled in a tai chi chih randomized controlled trial. *J. Cancer Surviv.* **2015**, *9*, 115-125.
20. Campo, R.A.; Agarwal, N.; LaStayo, P.C.; O'Connor, K.; Pappas, L.; Boucher, K.M.; Gardner, J.; Smith, S.; Light, K.C.; Kinney, A.Y. Levels of fatigue and distress in senior prostate cancer survivors enrolled in a 12-week randomized controlled trial of Qigong. *J. Cancer Surviv.* **2014**, *8*, 60-69.
21. Chen, Z.; Meng, Z.; Milbury, K.; Bei, W.; Zhang, Y.; Thornton, B.; Liao, Z.; Wei, Q.; Chen, J.; Guo, X.; et al. Qigong improves quality of like in women undergoing radiotherapy for breast cancer: results of a randomized clinical trial. *Cancer* **2013**, *119*, 1690-1698, doi:10.1002/cncr.27904.
22. Lam, S.W.Y. *A randomized, controlled trial of Guolin qigong in patients receiving transcatheter arterial chemoembolisation for unresectable hepatocellular carcinoma*; Master dissertation, The University of Hong Kong, Hong Kong, China, **2004**.
23. Larkey, L.K.; Roe, D.J.; Weihs, K.L.; Jahnke, R.; Lopez, A.M.; Rogers, C.E.; Oh, B.; Guillen-Rodriguez, J. Randomized Controlled Trial of Qigong/Tai Chi Easy on Cancer-Related Fatigue in Breast Cancer Survivors. *Ann. Behav. Med.* **2015**, *49*, 165-176.
24. Loh, S.Y.; Lee, S.Y.; Murray, L. The Kuala Lumpur Qigong trial for women in cancer survivorship phase-efficacy of a three-arm RCT to improve QOL. *Asian Pac. J. Cancer Prev.* **2014**, *15*, 8127-8134.
25. Zeng, Y.; Luo, T.; Xie, H.;,Huang, M.; Cheng, A.S.K. Health benefits of qigong or tai chi for cancer patients: a systematic review and meta-analyses. *Complement Ther. Med.* **2014**, *22*, 173-186, doi:10.1016/j.ctim.2013.11.010.
26. Oh, B.; Butow, P.N.; Mullan, B.A.; Clarke, S.J.; Beale, P.J.; Pavlakis, N.; Lee, M.S.; Rosenthal, D.S.; Larkey, L.; Vardy, J. Effect of medical Qigong on cognitive function, quality of life, and a biomarker of inflammation in cancer patients: a randomized controlled trial. *Support. Care Cancer* **2012**, *20*, 1235-1242, doi:10.1007/s00520-011-1209-6.
27. Oh, B.; Butow, P.; Mullan, B.; Clarke, S.; Beale, P.; Pavlakis, N.; Kothe, E.; Lam, L.; Rosenthal, D. Impact of medical Qigong on quality of life, fatigue, mood and inflammation in cancer patients: a randomized controlled trial. *Ann. Oncol.* **2010**, *21*, 608-614, doi:10.1093/annonc/mdp479.

For a free access, informational video on Qigong for cancer care, goto: http://theqigongnetwork.com.
